# Reassessing cortical reorganization in the primary sensorimotor cortex following arm amputation

**DOI:** 10.1093/brain/awv161

**Published:** 2015-06-13

**Authors:** Tamar R. Makin, Jan Scholz, David Henderson Slater, Heidi Johansen-Berg, Irene Tracey

**Affiliations:** 1 Oxford Centre for Functional Magnetic Resonance Imaging of the Brain (FMRIB Centre), Nuffield Department of Clinical Neurosciences, University of Oxford, Oxford OX3 9DU, UK; 2 The Hospital for Sick Children, 555 University Avenue, Toronto M5G 1X8, Ontario, Canada; 3 Oxford Centre for Enablement, Nuffield Orthopaedic Centre, Oxford OX3 7HE, UK; 4 Nuffield Division Anaesthetics, Nuffield Department of Clinical Neurosciences, University of Oxford, Oxford OX3 9DU, UK

**Keywords:** pain, plasticity, amputees, functional MRI, phantom pain

## Abstract

The role of cortical activity in generating and abolishing chronic pain is increasingly emphasized in the clinical community. Perhaps the most striking example of this is the maladaptive plasticity theory, according to which phantom pain arises from remapping of cortically neighbouring representations (lower face) into the territory of the missing hand following amputation. This theory has been extended to a wide range of chronic pain conditions, such as complex regional pain syndrome. Yet, despite its growing popularity, the evidence to support the maladaptive plasticity theory is largely based on correlations between pain ratings and oftentimes crude measurements of cortical reorganization, with little consideration of potential contributions of other clinical factors, such as adaptive behaviour, in driving the identified brain plasticity. Here, we used a physiologically meaningful measurement of cortical reorganization to reassess its relationship to phantom pain in upper limb amputees. We identified small yet consistent shifts in lip representation contralateral to the missing hand towards, but not invading, the hand area. However, we were unable to identify any statistical relationship between cortical reorganization and phantom sensations or pain either with this measurement or with the traditional Euclidian distance measurement. Instead, we demonstrate that other factors may contribute to the observed remapping. Further research that reassesses more broadly the relationship between cortical reorganization and chronic pain is warranted.

## Abstract (precis)

The brain’s ability to reorganise itself is key to our recovery from injuries, but the subsequent mismatch between old and new organisation may lead to pain. Makin *et al.* argue against this ‘maladaptive plasticity’ theory by showing that phantom pain in upper limb amputees is independent of cortical remapping.

## Graphical abstract

**Figure:**
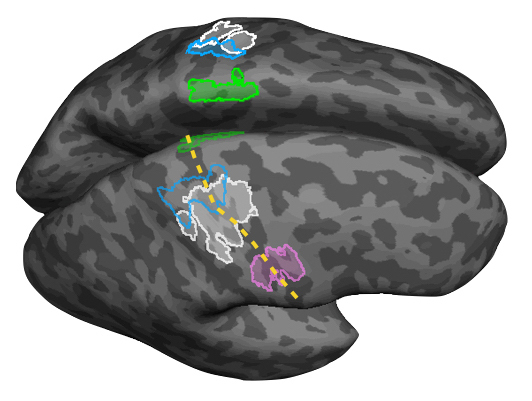
The brain’s ability to reorganise itself is key to our recovery from injuries, but the subsequent mismatch between old and new organisation may lead to pain. Makin *et al.* argue against this ‘maladaptive plasticity’ theory by showing that phantom pain in upper limb amputees is independent of cortical remapping.

## Introduction

Brain reorganization is a key mechanism that enables adjustment to novel situations and injuries, but it had also been suggested to have maladaptive consequences (Flor *et al.*, 2006). Amputation is a striking driver of plasticity, as it induces both sensory deprivation and altered behaviour. In monkeys, arm deafferentation drives massive cortical reorganization in the primary somatosensory cortex (SI), where the lower face representation takes over the cortical territory of the missing hand (Pons *et al.*, 1991; Jain *et al.*, 2008) (see Devor and Wall, 1978; Florence and Kaas, 1995; Kambi *et al.*, 2014 for reorganization in subcortical structures). In humans, remapping of lower face representation was shown to correlate with phantom limb pain (Flor *et al.*, 1995; Lotze *et al.*, 2001; Foell *et al.*, 2013). Subsequently, SI reorganization is increasingly assumed to play a key role in other syndromes of chronic pain (Maihöfner *et al.*, 2003; Nava and Röder, 2011; Gustin *et al.*, 2012), with important potential implications for designing clinical treatments (Moseley and Flor, 2012).

We recently reported that activity levels in the missing hands’ territory of amputees is not increased during lip movements (Makin *et al.*, 2013*b*). Instead, we found that phantom pain is associated with maintained structure and function during phantom hand movements. Nevertheless, this approach was not suitable for studying reorganization along the sensorimotor homunculus outside the missing hand territory. Sensorimotor reorganization in humans is typically measured as the Euclidian distance between the centre of gravity (CoG), or peak, in activity associated with facial touch (Flor *et al.*, 1995), or more recently lip movements (Lotze *et al.*, 2001; Foell *et al.*, 2013), and an ‘anchor’ (e.g. the mirror projection of the intact hand representation; Flor *et al.*, 1995). However, these measurements are taken across a folded cortical volume, and therefore do not respect anatomical barriers (e.g. white matter), or take into account the unique cortical morphology of individuals. As such, the physiological relevance of these measurements for cortical reorganization is potentially limited.

Here, we assessed remapping of sensorimotor lip representations using an unfolded model of the cortex, allowing us to measure surface-based cortical distances while considering individual cortical folding patterns (Maeda *et al.*, 2014) in 17 unilateral upper limb amputees and 21 intact controls. We found consistent shifts in lip representation along the homunculus contralateral to the missing hand in amputees (hereafter ‘deprived homunculus’) towards the hand area. However, this shift didn’t reflect full invasion of the lips into the hand territory as previously described, but rather a small local shift in the centre of gravity of the lips. This remapping was statistically independent of phantom pain ratings.

## Materials and methods

### Participants

Eighteen individuals with acquired unilateral upper limb amputation and varying degrees of phantom pain [mean age ± standard error of the mean (SEM) = 46 ± 3, six with absent right hand; Table 1] were recruited through the Oxford Centre for Enablement and Opcare. Twenty-two healthy controls, matched for handedness (seven left-hand dominant), age (41 ± 3 years) and education were also recruited. Procedures were in accordance with NHS national research ethics service approval (10/H0707/29), and written informed consent was obtained. Data from one amputee was discarded because of excessive head movements. One control and two amputees were discarded from subanalyses due to missing activity during feet (control) and phantom hand/arm (amputees) conditions (see below). The participants were studied intensively using a range of neuroimaging and behavioural tests and some of these data have been used to assess activity levels in the missing hand’s territory in previously published studies (Makin *et al.*, 2013*a*, *b*).

**Table 1:** Demographic and clinical details of the amputees, and individual imaging values

	Age	Age at amp.	Amputation level	Side/ dominant	PLS	PLP	Cause of amp.	Lips cortical shift (distance mm)	
					Mag./scan	Mag./scan/ave.			
								Euclid	Surface
**A01**	43	38	4	L/R	10/10	7/8/7	Trauma	6.25	8.21
**A02**	42	22	4	R/L	1.74/5	2.5/0/7	Nerve I*	6.66	−2.42
**A03**	21	18	4	R/L	8/9	3.33/0/5	Trauma	4.96	17.68
**A04**	46	37	2	L/R	3/2	2/0/1	Nerve I*	0.25	2.25
**A05**	48	20	1	R/R	10/7	4.5/1/9	Trauma	6.94	18.32
**A06**	58	11	2	R/R	1.2/6	1.75/0/2	Trauma	4.38	−4.64
**A07**	31	2	2	L/R	0/0	0/0/0	Trauma	4.07	14.82
**A08**	54	20	5	L/L	10/10	4/4/4	Trauma	−3.53	−7.50
**A09**	47	45	2	L/L	9/9	8/4/5	Tumour	4.64	4.23
**A10**	60	34	2	R/R	8/6	1/0/5	Trauma	10.20	13.29
**A11**	51	35	4	L/R	5/2	1.75/1/7	Infection	−5.94	−10.18
**A12**	47	19	2	L/R	4.5/6	4.5/4/4	Trauma*	4.01	32.81
**A13**	57	48	4	R/L	3.5/7	1.5/6/3	Infection	9.44	22.10
**A15**	22	18	5	L/R	10/10	1/0/2	Trauma	1.02	8.44
**A16**	43	33	4	L/R	2.67/4	2.33/0/6	Trauma	4.94	13.90
**A17**	50	28	4	L/R	5/2	3/0/4	Trauma	11.02	21.45
**A18**	52	45	4	L/R	1.33/0	0/0/0	Trauma	−14.90	−19.90

Amp. = amputation; Amputation levels: 1 = wrist, 2 = below elbow, 3 = through elbow, 4 = above elbow, 5 = through shoulder; Side = side of amputation; dominant = hand dominance prior to amputation (based on self-report), L = left, R = right; PLS = phantom limb sensations; Mag. = magnitude; scan = score of sensation vividness/pain intensity on scanning day; PLP = phantom limb pain; ave. = score of average pain; Nerve I = nerve injury; asterisk indicates potential partial spinal damage. Lip cortical shift is the difference between lip-to-feet cortical distances of the intact homunculus minus the deprived homunculus (positive values mark a medial shift). Euclid = Euclidian distance, measured in the folded brain. Surface = inflated surface analysis.

### Phantom sensations rating

Amputees rated intensities of phantom/stump pain and non-painful phantom sensations, using a 0–10 scale, as well as the frequency of these experiences, as follows: (i) intensity of worst pain/most vivid sensation experienced during the last week (or in a typical week involving such sensations); (ii) intensity of phantom pain on average over the last week (or in a typical week if last week was atypical); and (iii) current intensity/vividness of phantom pain and sensations, during scanning day. In addition, participants were asked to rate the intensity of an inventory of pain sensations (see Table 2 legend).

**Table 2:** Pearson’s partial coefficients of determination (r^2^) between phantom sensations and lip cortical distances in amputees**.**

		PLS		PLP						
		Mag.	Scan	Mag.	Scan	Ave.	Mechanic.	Thermal	Other	Stump pain
Lip-to-feet cortical distance (partial)	Euclidian (classical)	0.03	0.08	0.17	0.08	0.10	0.05	0.11	0.04	0.09
	Surface-base	0.01	0.02	0.03	0.10	0.00	0.01	0.03	0.00	0.04
Lip-to-hand cortical distance (partial)	Euclidian (classical)	0.12	0.04	0.12	0.17	0.04	0.06	0.00	0.01	0.01
	Surface-base	1.00	0.09	0.00	0.02	0.11	0.00	0.13	0.00	0.00
Lip-to-lip cortical distance (bivariate)	Euclidian (classical)	0.03	0.09	0.07	0.02	0.13	0.01	0.04	0.05	0.12
	Surface-base	0.02	0.04	0.04	0.04	0.03	0.10	0.00	0.01	0.06

To account for interindividual variation in structural and functional anatomy, cortical distances of the intact hand were included as a control variable (see Supplementary Table 2 for bivariate correlations). This table demonstrates that none of the phantom sensations and pain ratings significantly explains variation in lip-to-feet cortical distances, even when wavering correction for multiple comparisons.

PLS = phantom limb sensations; Mag. = magnitude; Scan = score of pain intensity on scanning day; PLP = phantom limb pain; Ave. = average phantom pain (relates to ratings during a typical week in which phantom pain is present); Mechanic. = mechanical pain (relates to scaled intensity ratings for the items: pulsing/stabbing/cutting/pushing/pinching/squashing); Thermal = thermal pain (relates to scaled intensity ratings for the items: hot/burning/chilly/freezing); Other = other pain (relates to scaled intensity ratings for the items: pricking/tingling/itchy/electric current). Partial/bivariate relates to the correlations performed. Lip-to-lip cortical distance reflects the difference between lip-to-feet cortical distance of the deprived hemisphere, versus the intact hemisphere.

‘Pain magnitude’ was calculated by dividing pain intensity by frequency (1, all the time; 2, daily; 3, weekly; 4, several times per month; and 5, once or less per month). An analogous measure was obtained for vividness of non-painful phantom sensations. See Table 1 for individual ratings and Supplementary Table 1 for dependencies between these various measurements.

### Functional MRI sensorimotor task

We used an active motor paradigm, similar to previous studies of reorganization and phantom pain (Lotze *et al.*, 2001; MacIver *et al.*, 2008; Foell *et al.*, 2013). In different conditions, participants were visually instructed to flex and extend their fingers, elbows, toes or smack their lips, resulting in six conditions: left/right hand; left/right arm, feet and lips. The protocol comprised of alternating 12-s periods of movement and ‘rest’, with each condition repeated four times, in a counterbalanced manner. It was stressed to the amputees that they should attempt to perform actual phantom hand movements, rather than imagined movements (see Makin *et al.*, 2013*b* for further details).

### Functional MRI data analysis

**Figure 1:**
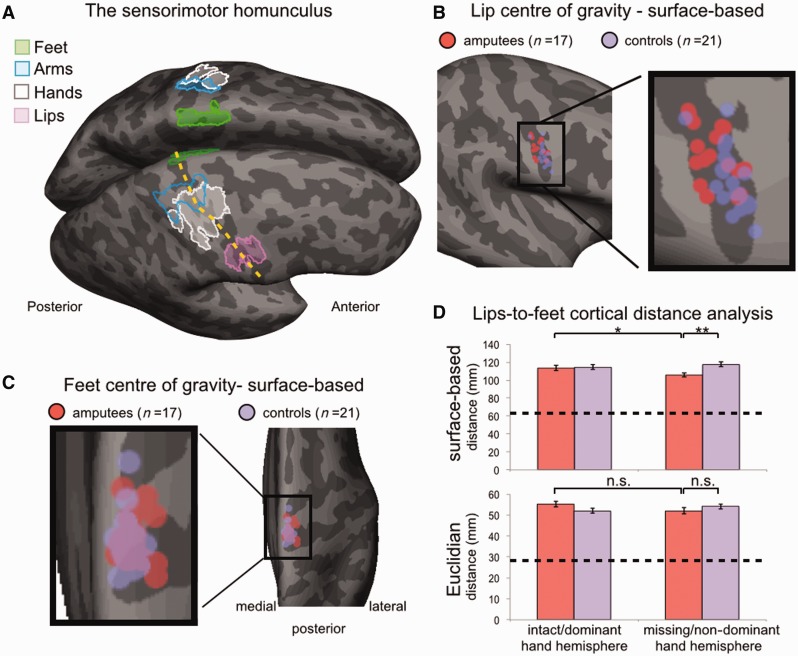
**Shifted lip representation in amputees.** (**A**) An illustration of the human sensorimotor homunculus, projected on a cortical surface map. Coloured contours delineate the boundaries of clusters activated during execution of movements using the feet (green), arms (blue), hands (white), and lips (pink) in controls. The hand representation is located approximately half way between the feet (medial) and lips (lateral). The yellow line indicates the central sulcus. The image was adapted from Makin *et al.* (2013*a*). (**B** and **C**) CoG of surface-based lip (**B**) and feet (**C**) representation for amputees (red) and controls (purple) in the central sulcus contralateral to the missing (amputees) or non-dominant hand (controls), projected on an averaged brain. While the amputees’ lip representations showed a medial displacement compared with the controls, the feet representation was largely overlapping (**D**) means (± SEM) of amputees (red) and controls (purple) lip-to-feet distance for each hemisphere, using the surface-based approaches (*top*) and the traditional (Euclidian distance in the folded brain, *bottom*). The dotted line shows the position of the hand (in the controls’ non-dominant hemisphere) along the homunculus. The surface-based approach showed a clear cortical shift in the lip representation along the deprived homunculus in amputees. However, note that the lip representation did not invade the hand area. Significance of planned comparisons (two-tailed *t*-tests) are denoted as follows: *P < 0.05; **P < 0.01. n.s. = non-significant (*P* > 0.05).

MRI data acquisition, preprocessing and preliminary analysis followed standard procedures, as detailed in the Supplementary material. Spatial resolution was 3 mm isotropic; temporal resolution was 2000 ms, with a total of 300 whole-brain samples (volumes). All functional MRI analysis was carried in individual’s native anatomical space. Functional data were processed using FSL FMRIB’s expert analysis tool (FEAT, version 5.98), using a Gaussian kernel of full-width at half-maximum of 2.5 mm for spatial smoothing. Task-based statistical parametric maps were computed for each condition using a voxel-based general linear model (GLM) based on the gamma function of the experimental time course and its temporal derivatives. To minimize any potential contribution of secondary somatosensory cortex to lip clusters, body-part-specific representation was identified using a contrast between each of the body parts and feet movements (whereas feet were compared to baseline). We note that similar results to those reported in Fig. 1 were also identified when lip activation maps were contrasted against baseline.

The active task involved both motor and somatosensory elements (e.g. resulting from contact between body parts or with clothes during movement execution, proprioceptive inputs, etc) and was designed to activate the primary somatosensory, as well as the primary motor cortices (SI and M1, respectively). While SI contains detailed body maps, M1 topography is relatively crude (Schieber, 2001; Graziano and Aflalo, 2007). Indeed, as can be seen in Fig. 1B, lip activations were centred on the central sulcus, suggesting that the resulting lip-specific representations had a strong somatosensory component. It should be noted that due to partial sampling, which is a consequence of the standard spatial resolution and smoothing applied, it is impossible to reliably dissociate SI and M1 contributions to the resulting clusters. Representations resulting from this task were therefore termed ‘sensorimotor’. However, to verify that the results are not restricted by the inclusion of M1 activation, the analysis reported in Fig. 1 was repeated while excluding the precentral gyrus, with comparable results.

### Cortical distances analysis

Automated reconstruction and segmentation of individual subjects’ T_1_ scans into surface mesh representations were carried out using FreeSurfer (http://surfer.nmr.mgh.harvard.edu/). The surface mesh of each cerebral hemisphere was inflated to a sphere while minimizing distortions to facilitate registration and maintain individual differences in cortical topology (Dale *et al.*, 1999). The body-part-specific statistical parametric maps were registered and projected to the individual sphere mesh. To allow us to specifically focus on topographic shifts in body-part representation, individual maps were masked by FreeSurfer's pre- and postcentral gyrus labels. Maps were thresholded by a false discovery rate (FDR) of q < 0.05 and a minimal use area of 100 mm^2^. The resulting maps were visually inspected to verify that the clusters were located along the sensorimotor strip. To minimize the potential contribution of neighbouring sensorimotor representations beyond the homunculus, clusters centred in the secondary somatosensory cortex and in the posterior parietal cortex surviving the contrasting and masking procedures were discarded from this analysis (see Makin *et al.*, 2015 for a study of whole-brain reorganization in amputees). As the aim of this analysis was simply to locate the spatial position of activation centres, the threshold criteria were waived when clusters did not survive the thresholding criteria (feet 2/3; lips 2/1; hands 1/0; arms 2/1 for amputees/controls, respectively). A CoG approach was applied to identify the location of each cluster. This approach was previously proven to be reliable across sessions, independently of functional MRI procedures (e.g. thresholding, clustering) for hand and lip movement representations (Fesl *et al.*, 2008). The CoG of a surface cluster was defined as the average position of all its vertices on the sphere. Therefore, the distance between the CoG was defined as the shortest distance between the points along the surface of the sphere (i.e. the great circle distance). Distances between the feet CoG and each of the body part representations were measured for each participant bilaterally. In addition, distances were measured for each hemisphere between the centres of gravity of hand and lip clusters, and between lip clusters CoGs and the intercept of the central sulcus with the medial wall (as specified manually by the experimenter). These measurements of lip distances from these various anchors strongly correlated with each other (all r’s > 0.74, all *P*-values <0.001), even when accounting for differences in brain size (by controlling for hand-to-feet distances). The clusters from the surface analysis were further projected back to a folded brain, and the CoG of a 3D cluster was defined as the average position of the voxels in each axis. The 3D distance was defined as the Euclidian distance across the three axes (Lotze *et al.*, 2001).

We also devised a preliminary analysis paradigm to visualize the spatial distribution of lip representation along the central sulcus. This analysis and (null) results are detailed in the Supplementary material and in Supplementary Fig. 2.

### Statistical comparisons

Statistical analysis was carried with SPSS version 22. Cortical distances along the deprived homunculus in amputees were compared with the intact homunculus in amputees and the hemisphere contralateral to the non-dominant hand in controls, initially using a mixed-model ANOVA (accounting for both hemispheres and groups) and then using *a priori* planned comparisons using independent-samples or paired two-tailed *t*-tests.

Another common measurement for assessing lip reorganization is made by comparing the ipsilateral and contralateral lip representation (Lotze *et al.*, 1999, 2001; Karl *et al.*, 2001; Foell *et al.*, 2013). In an effort to respect individuals’ unique brain topology, rather than ‘flipping’ brain representations across hemispheres (as previously practiced) we calculated the difference of lip-to-feet distance across the two hemispheres for each individual participant (Table 1).

To account for interindividual variations in structural and functional anatomy, correlations with phantom sensations and pain were assessed using a two-tailed Pearson partial correlation test, with distances in the deprived homunculus as the variable of interest, and the intact homunculus distances as the control variable (Table 2, see Supplementary Table 2 for bivariate correlations). As none of the phantom sensation or pain measurements were independently shown to underlie lip remapping, and as these measurements were interrelated, we next selected a subset of the phantom sensation and pain ratings, to feed into a multiple regression analysis, as described below.

To explore the potential contribution of phantom sensation and pain alongside other clinical and behavioural parameters that might associate with lip remapping, we used a backward-elimination linear regression. The following independent variables were added to the regression: (1–2) phantom sensations and pain magnitude (accounting for both intensity and frequency of experience); (3) averaged phantom pain intensity; (4) extent of residual arm (stump) usage, using motor activity questionnaires (as validated in Makin *et al.*, 2013*a*); (5) intact hand dexterity, measured using the pegboard task (Otten *et al.*, 2012); (6) tactile acuity of the intact index finger, measured using the grating orientation task (Bleyenheuft and Thonnard, 2007); (7) mouth and chin usage, measured using a customised questionnaire; (8) age at which amputation occurred; and (9) total brain volume, measured based on the anatomical scan. (See Supplementary Table 4 for bivariate correlations with lip reorganization and Supplementary material for details about assessment of the behavioural parameters). One participant was excluded from the tactile acuity analysis because of difficulties with task performance. Two further amputees didn’t complete the mouth usage questionnaire. The regression proceeds by considering different combinations of independent variables and eliminating the variable that explains least variance at each step. For each model, an r^2^-value quantifies the variance explained by the model and an F-value quantifies the model significance, which takes into account the number of independent variables in order to favour more simple models.

## Results

Lip mapping was initially measured as the cortical distance between the CoG of lip-selective (Fig. 1B) and feet (Fig. 1C) activations. Using the surface-based approach, we identified reliable lip reorganization in amputees, as reflected in a significant interaction between hemisphere (deprived, intact) and group (amputees, controls) [*F*(1,36) = 7.16, *P* = 0.011; Fig. 1D]. Lip distance was significantly shorter in the amputees’ deprived homunculus, compared with both their intact homunculus, [*t*(16)=2.37, *P* = 0.031] and controls’ non-dominant hand homunculus [*t*(36) = 3.11, *P* = 0.004]. This confirms remapping (i.e. shorter lip-to-feet distances) in the amputees’ deprived homunculus. On average, lips in the deprived homunculus were shifted medially by 7.8 mm, compared to the intact homunculus (Table 1, see also Supplementary Fig. 2).

We also applied the ‘traditional’ Euclidian distance approach in the folded brain (Gustin *et al.*, 2012; Foell *et al.*, 2013). Cortical distances measured with this approach correlated significantly with the surface-based values [r(36) = 0.61/0.79 for intact/deprived hemispheres, *P* < 0.002, even when accounting for differences in brain size (by controlling for hand-to-feet distances in the intact/dominant hand hemisphere)]. Accordingly, a similar interaction to that described above was identified in the folded brain [*F*(1,36) = 6.31, *P* = 0.017]. However, the subsequent planned comparisons were not significant, suggesting that the interaction wasn’t entirely driven by reduced cortical distances in the deprived hemisphere of amputees (Fig. 1D). On average, lip representation was shifted by 3.2 mm in the folded deprived cortex compared to the intact homunculus. This shift is substantially smaller than previously reported (e.g. averaged shifts of 15 mm; Foell *et al.*, 2013, see also Flor *et al.*, 1995).

We also used the surface-based approach as described above to study stability of hand and arm representation following amputation. Consistent with our previous reports (Makin *et al.*, 2013*a*, *b*), cortical distances in the deprived homunculus did not vary for residual arm (Supplementary Table 3) or phantom hand representations (Supplementary Fig. 1). This latter finding allowed us to further examine cortical distances directly between lip and phantom hand representation on the cortical surface [note that the traditional Euclidian analysis showed significant lateral shifts of the intact hand of amputees, compared to controls; *t*(35) = 2.639, *P* = 0.012]. Lip-to-phantom hand distances (or non-dominant hand, in controls) correlated strongly both with lip-to-feet distances (as described above), and with lip-to-medial wall distances [r(37) = 0.82/0.74, respectively; *P* < 0.001]. Accordingly, we identified significant group difference using the phantom hand anchor, [*t*(35) = −2.149, *P *= 0.039], reflecting shorter distances in amputees (mean distance 59 mm) compared with controls (63 mm), further confirming small medial shifts of lip representation in amputees’ deprived hemisphere.

Next, we examined the role of phantom sensation and pain in driving the observed lip remapping. Even when wavering correction for multiple comparisons, no single variable showed significant correlation with any of the lip cortical distance parameters (i.e. lip-to-feet, lip-to-hand or lip-to-lip distances; see Table 2 and Supplementary Table 2). To test whether any combination of factors relating to phantom pain, sensation or other clinical variables could account for the remapping, exploratory backward elimination regressions were run, using our original measurement (lip-to-feet distances). For surface-based distances, the most parsimonious model relied on tactile acuity and dexterity of the intact hand as the independent variable [R^2 ^= 0.65, *F*(2,11) = 10.14, *P* = 0.003; adjusted R^2 ^= 0.58, Supplementary Table 5; see Supplementary material for information about assessment of intact hand dexterity and acuity]. For the traditional (folded brain) analysis, most variance was explained solely based on brain size [R^2 ^= 0.41, *F*(1,12) = 8.33, *P* = 0.014; adjusted R^2 ^= 0.36, Supplementary Table 6]. No significant model fit was found using the same parameters for surface-based lip-to-feet distances in the intact homunculus. This demonstrates that no single clinical factor explains changes in cortical distances and that a combination of behavioural and methodological factors should be considered when interpreting these measurements.

## Discussion

Using a surface-based approach, which takes into account individual brain morphology, we identified reliable lip remapping in the deprived homunculus of amputees. This shift may reflect invasion of the lip representation towards the missing hand cortex, as described in seminal electrophysiology studies (Pons *et al.*, 1991). However, this shift was only partial (8 mm), and did not reflect full invasion of the lips into the hand territory (which is located some 63 mm from the lips in the controls’ homunculus, see also Supplementary Fig. 2 for complementary analysis). This result is consistent with our previous findings, showing maintained activity of the phantom hand in the missing hands’ territory of amputees (Makin *et al.*, 2013*b*) (Supplementary Fig. 1).

Contrary to previous studies and despite our relatively large sample size, we were unable to identify any statistical relationship between cortical reorganization and phantom sensations or pain. This could be attributed to differences in the underlying assumptions of the different experimental approaches. For example, in their seminal paper, Flor *et al.* (1995) used electrical source estimates of lip and cheek foci, distances were measured with respect to the intact hand, and phantom pain was assessed with respect to pain intensity and suffering. To bridge this gap, it is important that further studies are carried out, while taking into consideration the methodological and conceptual constraints highlighted here, namely the usage of physiologically realistic measurements of reorganization and attention to other clinical factors that could be driving brain plasticity. Our current results are consistent with a recent study in patients with carpal tunnel syndrome that identified correlations between surface-based SI reorganization and paraesthesia severity, but no correlation with pain (Maeda *et al.*, 2014). The view that multiple factors may shape reorganization is also in accordance with our recent findings for use-dependent plasticity in the deprived cortex of amputees (Makin *et al.*, 2013*a*; Hahamy *et al.*, 2015). Therefore, our results call for a reassessment of maladaptive plasticity theories ascribing a specific causal role to cortical remapping in driving chronic pain and the associated treatments targeting this remapping to alleviate pain.

## Abbreviations

CoG: centre of gravity
SI: primary somatosensory cortex

## References

[awv161-B1] Bleyenheuft Y Thonnard J Tactile spatial resolution measured manually: a validation study. Somatosensory & Motor. 2007; Somatosens Mot Res; 24: 111–4. 10.1080/08990220701496639 17853056

[awv161-B2] Dale AM Fischl B Sereno MI Cortical surface-based analysis. I. Segmentation and surface reconstruction. Neuroimage 1999; 9: 179–94. 9931268 10.1006/nimg.1998.0395

[awv161-B3] Devor M Wall PD Reorganisation of spinal cord sensory map after peripheral nerve injury. Nature 1978; 276: 75–6. 570248 10.1038/276075a0

[awv161-B4] Fesl G Braun B Rau S Wiesmann M Ruge M Is the center of mass (COM) a reliable parameter for the localization of brain function in fMRI? Eur Radiol 2008; 18; 1031–7. 18228024 10.1007/s00330-008-0850-z

[awv161-B5] Flor H Elbert T Knecht S Wienbruch C Pantev C Birbaumer N Phantom-limb pain as a perceptual correlate of cortical reorganization following arm amputation. Nature 1995; 375: 482–4. 7777055 10.1038/375482a0

[awv161-B6] Flor H Nikolajsen L Staehelin Jensen T Phantom limb pain: a case of maladaptive CNS plasticity? Nat Rev Neurosci 2006; 7: 873–81. 17053811 10.1038/nrn1991

[awv161-B7] Florence SL Kaas JH Large-scale reorganization at multiple levels of the somatosensory pathway follows therapeutic amputation of the hand in monkeys. J Neurosci 1995; 15: 8083–95. 8613744 10.1523/JNEUROSCI.15-12-08083.1995 PMC6577958

[awv161-B8] Foell J Bekrater-Bodmann R Diers M Flor H Mirror therapy for phantom limb pain: Brain changes and the role of body representation. Eur J Pain 2013; 18: 729–39. 24327313 10.1002/j.1532-2149.2013.00433.x

[awv161-B9] Graziano MSA Aflalo TN Mapping behavioral repertoire onto the cortex. Neuron 2007; 56: 239–51. 17964243 10.1016/j.neuron.2007.09.013

[awv161-B10] Gustin SM Peck CC Cheney LB Macey PM Murray GM Henderson LA Pain and plasticity: is chronic pain always associated with somatosensory cortex activity and reorganization? J Neurosci 2012; 32: 14874–84. 23100410 10.1523/JNEUROSCI.1733-12.2012 PMC6704842

[awv161-B11] Hahamy A Sotiropoulos SN Henderson Slater D Malach R Johansen-Berg H Makin TR Normalisation of brain connectivity through compensatory behaviour, despite congenital hand absence. eLife 2015; 4. 10.7554/eLife.04605 PMC4281879 25562885

[awv161-B12] Jain N Qi H-X Collins CE Kaas JH Large-scale reorganization in the somatosensory cortex and thalamus after sensory loss in macaque monkeys. J Neurosci 2008; 28: 11042–60. 18945912 10.1523/JNEUROSCI.2334-08.2008 PMC2613515

[awv161-B13] Kambi N Halder P Rajan R Arora V Chand P Arora M Large-scale reorganization of the somatosensory cortex following spinal cord injuries is due to brainstem plasticity. Nat Commun 2014; 5: 3602. 24710038 10.1038/ncomms4602

[awv161-B14] Karl A Birbaumer N Lutzenberger W Cohen LG Flor H Reorganization of motor and somatosensory cortex in upper extremity amputees with phantom limb pain. J Neurosci 2001; 21: 3609–18. 11331390 10.1523/JNEUROSCI.21-10-03609.2001 PMC6762494

[awv161-B15] Lotze M Flor H Grodd W Larbig W Birbaumer N Phantom movements and pain. An fMRI study in upper limb amputees. Brain 2001; 124: 2268–77. 11673327 10.1093/brain/124.11.2268

[awv161-B16] Lotze M Grodd W Birbaumer N Erb M Huse E Flor H Does use of a myoelectric prosthesis prevent cortical reorganization and phantom limb pain? Nat Neurosci 1999; 2: 501–2. 10448212 10.1038/9145

[awv161-B17] MacIver K Lloyd DM Kelly S Roberts N Nurmikko T Phantom limb pain, cortical reorganization and the therapeutic effect of mental imagery. Brain 2008; 131: 2181–91. 18567624 10.1093/brain/awn124 PMC2494616

[awv161-B18] Maeda Y Kettner N Holden J Lee J Kim J Cina S Functional deficits in carpal tunnel syndrome reflect reorganization of primary somatosensory cortex. Brain 2014; 137: 1741–52. 24740988 10.1093/brain/awu096 PMC4032104

[awv161-B19] Maihöfner C Handwerker HO Neundörfer B Birklein F Patterns of cortical reorganization in complex regional pain syndrome. Neurology 2003; 61: 1707–15. 14694034 10.1212/01.wnl.0000098939.02752.8e

[awv161-B20] Makin TR Cramer AO Scholz J Hahamy A Henderson Slater D Tracey I Deprivation-related and use-dependent plasticity go hand in hand. eLife 2013a; 2. 10.7554/eLife.01273 PMC3823186 24220510

[awv161-B21] Makin TR Filippini N Duff EP Slater DH Tracey I Johansen-Berg H Network-level reorganisation of functional connectivity following arm amputation. Neuroimage 2015 10.1016/j.neuroimage.2015.02.067 PMC4461307 25776216

[awv161-B22] Makin TR Scholz J Filippini N Henderson Slater D Tracey I Johansen-Berg H Phantom pain is associated with preserved structure and function in the former hand area. Nat Commun 2013b; 4: 1570. 23463013 10.1038/ncomms2571 PMC3615341

[awv161-B23] Moseley GL Flor H Targeting cortical representations in the treatment of chronic pain: a review. Neurorehabil Neural Repair 2012; 26: 646–52. 22331213 10.1177/1545968311433209

[awv161-B24] Nava E Röder B Adaptation and maladaptation insights from brain plasticity. Prog Brain Res 2011; 191: 177–94. 21741552 10.1016/B978-0-444-53752-2.00005-9

[awv161-B25] Otten ML Mikell CB Youngerman BE Liston C Sisti MB Bruce JN Motor deficits correlate with resting state motor network connectivity in patients with brain tumours. Brain 2012; 135: 1017–26. 22408270 10.1093/brain/aws041 PMC3326259

[awv161-B26] Pons TP Garraghty PE Ommaya AK Kaas JH Taub E Mishkin M Massive cortical reorganization after sensory deafferentation in adult macaques. Science 1991; 252: 1857–60. 1843843 10.1126/science.1843843

[awv161-B27] Schieber MH Constraints on somatotopic organization in the primary motor cortex. J Neurophysiol 2001; 86: 2125–43. 11698506 10.1152/jn.2001.86.5.2125

